# Serum Calprotectin is Indicating Clinical and Ultrasonographic Disease Activity in Rheumatoid Arthritis, even with Normal C-Reactive Protein Levels

**DOI:** 10.31138/mjr.32.1.56

**Published:** 2021-02-15

**Authors:** Murat Torgutalp, Mucteba Enes Yayla, Didem Sahin Eroglu, Ayse Bahar Kelesoglu Dincer, Emine Uslu Yurteri, Ilyas Ercan Okatan, Serdar Sezer, Emine Gozde Aydemir Guloksuz, Ebru Us, Tahsin Murat Turgay, Gulay Kinikli, Askin Ates

**Affiliations:** 1 Division of Rheumatology, Department of Internal Medicine, and; 2 Department of Medical Microbiology, Ankara University Faculty of Medicine, Ankara, Turkey

**Keywords:** Acute phase, biomarker, calprotectin, rheumatoid arthritis, ultrasonography, ultrasound

## Abstract

**Objective:**

Calprotectin is an inflammatory biomarker which assesses disease activity in rheumatoid arthritis (RA). The objective of this study was to test whether serum calprotectin is associated with clinical and ultrasonographic disease activity in patients with RA, and to analyse its predicting value for disease activity evaluation despite normal C-Reactive protein (CRP) levels.

**Methods:**

We included 80 patients with RA and 30 healthy subjects. Patients were examined clinically and by ultrasound, (US7 score) along with laboratory parameters (calprotectin, CRP, erythrocyte sedimentation rate [ESR]). Disease activity scores (DAS28) were calculated to assess disease activity. Firstly, patients were divided into four subgroups according to the DAS28-ESR (high, moderate, low disease activity, and remission), then into two subgroups; group-1 (DAS-28≤3.2) and group-2 (DAS28>3.2). The predicting value of calprotectin for disease activity in patients with normal CRP was analysed with univariate and multivariate analysis and receiver operating characteristic curves.

**Results:**

Calprotectin levels were higher in RA patients than controls (96.3±45.9 ng/ml, 54.7±50.0 ng/ml, respectively; p<0.001). Calprotectin levels were 74.8±45.5 ng/ml in group-1 (n=37) and 114.7±37.9 ng/ml in group-2 (n=43) (p<0.001). In univariate analyses, calprotectin was significantly correlated with clinical, laboratory, and ultrasound parameters (p<0.05), and was a better predictor of power doppler synovitis than CRP in multivariate analysis (OR=1.014; 95%CI 1.002–1.027; p=0.024). The discriminatory capacity for calprotectin to distinguish ultrasonographically active disease in patients with normal CRP levels using AUC was 0.75 (95%CI 0.56–0.90, p=0.023).

**Conclusions:**

Calprotectin represents disease activity, even in patients who are clinical and ultrasonographical active but have normal CRP levels.

## INTRODUCTION

Rheumatoid arthritis (RA) is an autoimmune disease characterised by the inflammation of joints, has a complex pathogenesis, and follows a chronic course by synovial joint inflammation, erosions, irreversible joint damage, and subsequent functional loss.^1,2^ Currently, the Disease Activity Score (DAS) and indexes such as the Clinical Disease Activity Index (CDAI) and the Simplified Disease Activity Index (SDAI) are used to evaluate disease activity in RA.^3,4^ Tender joint count (TJC) and swollen joint count (SJC) of 28 joints, erythrocyte sedimentation rate (ESR) or C-reactive protein (CRP) levels as laboratory parameters, and patient’s global assessment by using the visual analog scale need to be known when defining the disease activity with aforementioned indexes.

Calprotectin is a heterocomplex of the S100A8/A9 proteins and also called as a myeloid-related protein 8/14.^5^ This protein has been shown to be elevated in a variety of autoimmune inflammatory diseases and is classified as a damage-related molecule. Also, it is used to assess disease activity in inflammatory bowel disease.^6,7^ Calprotectin is a pro-inflammatory protein that binds to granulocytes, monocytes, and macrophages, and induces the release of cytokines like interleukin-1 and -6 and tumour necrosis factor-alpha, which are important in RA pathogenesis.^8^ Calprotectin is released not only from granulocytes and macrophages in circulation, but also from synovial tissue macrophages, activated leukocytes in the area of inflammation, and activated synovial cells.^9,10^ Therefore, serum calprotectin levels reflect local activity as well as systemic inflammatory activity.

Musculoskeletal ultrasonography (MSUS), a widely-used non-invasive imaging technique, is used to evaluate joint inflammation with a good sensitivity.^11^ Power Doppler (PD) identifies the synovial flow which shows elevated synovial vascularisation, and it discriminates active synovial inflammation from synovial hypertrophy.^12^ In addition, PD is a method that demonstrates disease activity more sensitively than other clinical tools.^13^ Moreover, PD synovitis is considered to be an indicator of continuing joint damage and clinical exacerbations, even in patients without any clinical and/or laboratory findings of disease activity.^14^ The US7 (7 joint ultrasound score), described by Backhaus et al., is validated, and the simplest and most practical method among the currently used ultrasonographic scoring systems.^15^ Hammer and colleagues compared ultrasonographic findings by using different numbers of joints (7, 12, 28, 44, and 78), and found that US7 and US78 scores had similar sensitivity when assessing disease activity in RA.^16^

Previous studies showed that serum calprotectin levels were correlated with disease activity and can predict therapy response in patients with RA.^17–19^ Several studies have described that calprotectin was better than CRP in indicating inflammation detected by ultrasound.^20–22^ However, there is limited evidence regarding the importance of calprotectin in indicating activity in RA patients with normal CRP levels.^21^ There is a need for a simple and rapid biomarker that accurately demonstrates disease activity and inflammation in RA patients. Therefore, the primary aim of this cross-sectional observational study was to investigate the relationship between serum calprotectin levels and commonly used laboratory, clinical, and ultrasonographic activity parameters. The further aims of the study were to establish the indicative value of calprotectin for PD activity and to analyse the clinical value of calprotectin in patients with normal CRP levels.

## MATERIALS AND METHODS

### Study groups

Eighty patients were selected from our rheumatology outpatient clinic in 6 months. All study subjects met the 2010 American College of Rheumatology/European League Against Rheumatism (ACR/EULAR) criteria for RA.^23^ Thirty healthy subjects, without a diagnosis of rheumatologic disease, kidney or liver diseases, active infections, or cancer, were included as a control group from the internal medicine outpatient clinic. All the selected participants gave informed written consent. The study was conducted according to the rules of the Helsinki Declaration and was approved by the local medical ethics committee of the University.

Clinical information such as demographic characteristics, disease duration, medications, morning stiffness, and smoking status were recorded for all patients. Physical examinations of joints for all patients were performed by their physicians during an outpatient clinic visit.

Disease activity was assessed by the DAS28, CDAI, and SDAI tools, and these scores were calculated using TJC, SJC, CRP and/or ESR, the patient global assessment by using the VAS, the care provider global health assessment.^3,4^ The patients were divided into four subgroups according to the DAS28-ESR score: stable or remission (DAS28-ESR < 2.6), low disease activity (LDA, 2.6 ≤ DAS28-ESR < 3.2), moderate disease activity (MDA, 3.2 ≤ DAS28-ESR < 5.1), and high disease activity (HDA, DAS28-ESR ≥ 5.1). For the main comparison, the study patients were classified according to their DAS28-ESR scores as group 1; DAS28-ESR < 3.2 and group 2; DAS28-ESR ≥ 3.2. The health assessment questionnaire (HAQ) was used for the evaluation of self-reported functional status measurement.

### Specimen collection and laboratory evaluation

Serum samples were collected on the same day when the MSUS examination was performed. Serum was obtained from centrifuged venous blood (5 ml) from the patients and the controls in a fasted state. The samples were centrifuged within 2 hours after taken and preserved at −80 °C until measurements. Calprotectin levels were tested by a commercially available enzyme-linked immunosorbent assay (ELISA), by following the assay kit instructions (Human Calprotectin ELISA Kit, Elabscience Biotechnology Co., China). The examination was performed using the automated biochemical analysers. The ESR was determined using the Westergren method (mm/h) and the CRP level was determined using the automatic immune rate nephelometry (mg/L). A CRP cutoff < 5 mg/L was accepted as normal according to the results of the local laboratory. The level of anti-citrullinated protein antibodies (ACPA) and rheumatoid factor was determined using standard ELISA kits.

### MSUS assessment

The ultrasonographic examination was carried out using high-sensitive ultrasound equipment Esaote Mylab 70 device (Esaote S.p.A., Genova, Italy), with a high frequency linear array probe (6–18 MHz). The pre-set settings were used to assess PD signal activity. Ultrasound assessments of seven joints were done by a two-year experienced sonographer (ABKD). The ultrasonographer was not informed about the clinical examination and laboratory findings of the participants. The German US7 score was applied for the MSUS examination.^15^ The 7 articular joints were: wrist, the second and third meta-carpophalangeal (MCP2 and MCP3) and proximal inter-phalangeal (PIP2 and PIP3) joints, and the second and fifth metatarsophalangeal (MTP2 and MTP5) joints of the dominantly affected hand and foot. Synovitis, tenosynovitis, and bone erosion of these joints were evaluated. Synovitis in the grayscale (GS) was scored semi-quantitatively (0 = absence, 1 = mild, 2 = moderate, 3 = severe synovitis), as follows: grade 1 = a small hypoechoic/anechoic line beneath the joint capsule; grade 2 = the joint capsule raised parallel to the joint area; and grade 3 = a strong expansion of the joint capsule.^24^ When synovitis was detected, further assessment for the existence of inflammation was done by using coloured Doppler.^25^ The Doppler signal was assessed into the three following categories: grade 0 = no intraarticular colour signal; grade 1 = a few spots of the blood flow signal; grade 2 = a continuous flow signal with an area greater than grade 1 and <50% of the joint cavity; and grade 3 = blood flow in more than 50% of the joint cavity. Tenosynovitis in the GS and bone erosions were classified as absent (0) or present (1). An overall GS and PD scores were calculated as the sum of GS synovitis (0–27), PD synovitis (0–39), GS tenosynovitis (0–7), and PD tenosynovitis (0–21). The synovitis, tenosynovitis, and respective flow signals were noted for seven joints. A score of zero in PD and GS synovitis was defined as ultrasound remission.

### Statistical analysis

Statistical evaluations were done by using the Statistical Package for the Social Sciences (SPSS) version 21 statistical software (SPSS, Chicago, IL, USA). The data were defined as mean ± standard deviation, unless stated otherwise. The variables were considered using analytical (Kolmogorov-Simirnov / Shapiro-Wilk’s test) and visual (histograms, probability plots) methods to control the normal distribution. The Chi-square or Fisher’s exact test was used to analyse proportions in diverse groups. Independent sample t-test (or Mann-Whitney U test as a nonparametric substitute) and ANOVA (or Kruskal Wallis test as a nonparametric substitute) were used to analyse the differences between extracted groups, followed by post hoc tests with Bonferroni correction. Spearman’s rank analyses were used to assess the correlations between serum calprotectin and other variables. Receiver-Operating Characteristic (ROC) curve analysis was used to evaluate the prediction capacity of calprotectin for the presence of inflammation. When a compelling cut-off value was detected, specificity and sensitivity were represented. For the multivariate analysis, potential factors described by univariate analyses have been inserted into the logistic regression model to define independent predictors of the presence of PD synovitis. We used Hosmer-Lemeshow goodness of fit statistics to evaluate model fit. A two-sided p value below 0.05 was accepted to show a significant statistical outcome.

## RESULTS

### General characteristics of patients with RA and controls

There were 80 patients with RA, predominantly women (n=63, 78.8 %), with a mean age of 57.2 ± 9.6 years. In the control group, there were 21 women (70 %), with a mean age of 53.9 ± 10.5. Age and sex distributions were statistically similar between the patients with RA and the control group (p=0.12 and p=0.34, respectively). Serum calprotectin levels were significantly higher in the patients with RA than the control group (96.35 ± 45.91 ng/ml vs 54.68 ± 50.04 ng/ml, p < 0.001 respectively).

The clinical, laboratory, and ultrasonographic features of the study group are presented in **Table 1**. During the examination, 11 patients were in remission (DAS28-ESR < 2.6), 26 patients had LDA, 34 patients had MDA, and 9 patients had HDA. Positive ACPA and RF levels were detected in 57 (71.3%) and 52 (65%) of RA patients, respectively. Among our patients, 52 (65%) patients never smoked, 11 (13.7%) were past smokers, and 17 (21.3%) were current smokers.

**Table 1.** Baseline characteristics, laboratory, and ultrasound values of 80 patients with RA^*^.

**Characteristics**	**Total (n=80)**	**Group 1 (n=37)**	**Group 2 (n=43)**	**p**^§^
Female Sex, n (%)	63 (78.8)	30 (81.1)	33 (76.7)	0.64
Age, years	57.2 ± 9.6	55.4 ± 9.4	58.8 ± 9.6	0.12
Disease duration, years	12.7 ± 8.3	13.2 ± 9.1	12.2 ± 7.6	0.61
BMI (kg/m^2^)	29.4 ± 5.7	28.4 ± 4.9	30.3 ± 6.2	0.11
Smoking status, n (%)				
Current smoker	17 (21.3)	10 (27)	7 (16.3)	0.50
Past smoker	11 (13.7)	5 (13.5)	6 (14)	
Never smoked	52 (65)	22 (59.5)	30 (69.7)	
csDMARDs, n (%)	55 (68.7)	25 (67.6)	30 (69.8)	0.83
bDMARDs, n (%)	46 (57.5)	21 (56.8)	25 (58.1)	0.90
Steroids, n (%)	46 (57.5)	24 (64.9)	22 (51.2)	0.22
Prednisolone dose, mg/d	8.03 ± 5.01	7.19 ± 3.78	8.95 ± 6.04	0.24
NSAIDs, n (%)	59 (73.7)	24 (64.9)	35 (81.4)	0.10
RF positivity, n (%)	57 (71.3)	26 (70.3)	31 (72.1)	0.86
ACPA positivity, n (%)	52 (65)	24 (64.9)	28 (65.1)	0.98
TJC	3.1 ± 2.6	1.9 ± 1.0	4.2 ± 3.0	***< 0.001***
SJC	1.0 ± 2.1	0.2 ± 0.4	1.7 ± 2.6	**0.001**
PGH, 0–100 mm	22.7 ± 15.9	13.7 ± 10.1	30.5 ± 15.9	***< 0.001***
Morning stiffness, minute	19.9 ± 23.2	13.8 ± 20.1	25.1 ± 24.7	***0.028***
VAS Pain, 0–100 mm	39.8 ± 24.7	29.5 ± 20.2	48.7 ± 25.0	***< 0.001***
ESR, mm/hour	29.1 ± 20	14.2 ± 7.2	42.0 ± 18.5	***< 0.001***
CRP, mg/dl	16.1 ± 21.7	8.2 ± 11.8	23.0 ± 25.7	***0.002***
DAS28-ESR score	3.60 ± 1.01	2.7 ± 0.4	4.3 ± 0.8	***< 0.001***
DAS28-CRP score	3.15 ± 0.95	2.5 ± 0.4	3.7 ± 0.9	***< 0.001***
CDAI score	8.1 ± 6.3	4.3 ± 2.1	11.4 ± 6.9	***< 0.001***
SDAI score	24.2 ± 25.4	12.4 ± 11.9	34.4 ± 29.4	***< 0.001***
HAQ score	0.40 ± 0.37	0.22 ± 0.23	0.55 ± 0.39	***< 0.001***
GS syn score (0–27)	2.93 ± 2.15	2.38 ± 1.74	3.40 ± 2.37	***0.034***
PD syn score (0–27)	1.98 ± 2.60	1.30 ± 1.82	2.56 ± 3.01	***0.029***
GS ten score (0–7)	0.95 ± 1.14	0.76 ± 1.09	1.12 ± 1.16	0.16
PD ten score (0–7)	0.78 ± 1.10	0.49 ± 0.84	1.02 ± 1.24	***0.029***
US7 total score	6.63 ± 6.18	4.92 ± 4.82	8.09 ± 6.87	***0.021***
Erosion score (0–14)	3.58 ± 2.43	3.27 ± 2.35	3.84 ± 2.50	0.30
Calprotectin, ng/ml	96.3 ± 45.9	74.8 ± 45.5	114.7 ± 37.9	***< 0.001***

* Group 1: DAS28-ESR < 3.2, Group 2: DAS28-ESR ≥ 3.2

§ All p values are between group 1 and group 2

ACPA: anti-citrullinated protein antibodies; BMI: body mass index; bDMARDs: biologic disease-modifying antirheumatic drugs; CDAI: clinical disease activity index; CRP C-reactive protein; csDMARDs: conventional synthetic disease-modifying antirheumatic drugs; DAS28: disease activity score in 28 joints; ESR: erythrocyte sedimentation rate; GS syn: gray scale synovitis; GS ten: gray scale tenosynovitis; HAQ: health assessment questionnaire; IQR interquartile range; NSAIDs: nonsteroidal anti-inflammatory drugs; PD syn: power doppler synovitis; PD ten: power doppler tenosynovitis; PGH: patient global health; RA: rheumatoid arthritis; RF: rheumatoid factor; SDAI: simplified disease activity index; SJC: swollen joint count; syn: synovitis; ten: tenosynovitis; TJC: tender joint count; US7: the 7-joint ultrasound score; VAS: visual analog scale.

Patients were divided into two groups according to their DAS28 scores (Group 1 ≤3.2 [remission and LDA] and group 2 >3.2 [MDA and HDA]). The ESR, CRP, TJC, SJC, HAQ, DAS28-CRP, SDAI and CDAI were significantly higher in group 2 (**Table 1**). There was no statistical difference between the two groups’ mean disease duration. There was no significant difference in the percentage of their smoking status between the two groups (p=0.50). Percentages of patients according to the usage of conventional synthetic disease-modifying antirheumatic drugs (csDMARDs), biologic-DMARDs (bDMARDs), steroids, and nonsteroidal anti-inflammatory drugs (NSAIDs) were similar between two groups (p values were; 0.83, 0.90, 0.22 and 0.10 respectively). There was no significant difference in the positivity of ACPA and RF between group 1 and group 2 (p=0.98 and 0.86, respectively).

### Serum calprotectin levels and disease activity assessment in patients with RA

**Figure 1.**
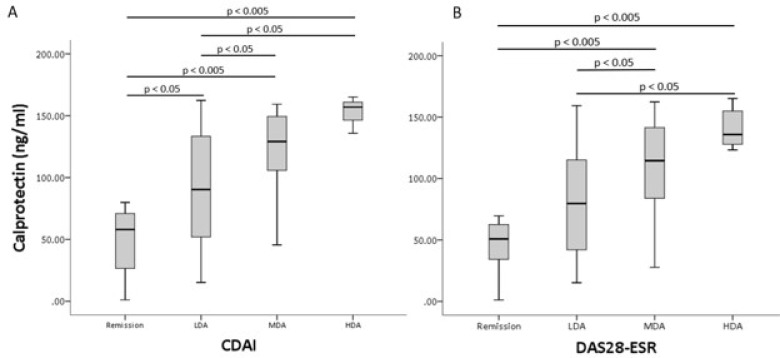
Associations between serum calprotectin levels and disease activity in patients with rheumatoid arthritis according to A) CDAI B) DAS28-ESR.

Calprotectin levels were shown according to disease activity subgroups determined by CDAI and DAS28 in **Figure 1**. Serum calprotectin levels in patients with HDA were significantly higher than those in the remission and LDA groups (p <0.005 and p <0.05, respectively). Calprotectin in patients with MDA was significantly higher than in patients with remission and LDA (p <0.005 and p <0.05, respectively). There was no significant difference in the levels of this marker between MDA and HDA groups.

Serum calprotectin levels were higher in RF negative than in RF positive patients; however, these differences were not statistically significant (105.6±47.1 vs 92.5±5.3 ng/ml, respectively, p=0.25). There was also no statistical difference in calprotectin levels between ACPA negative and ACPA positive patients (108±44.4 vs 89.9±45.9 ng/ ml, respectively, p=0.09).

When the serum calprotectin levels were compared between patients using and not using bDMARDs, no significant difference was found between the two groups (99.2 ± 44.3 vs 92.4 ± 48.4 ng/ml, respectively, p = 0.52). Similarly, there was no difference between patients who received steroids and those who did not (99.0 ± 44.3 vs 92.6 ± 48.6 ng/ml, respectively, p = 0.54).

### Disease activity assessments and US7 score

**Figure 2.**
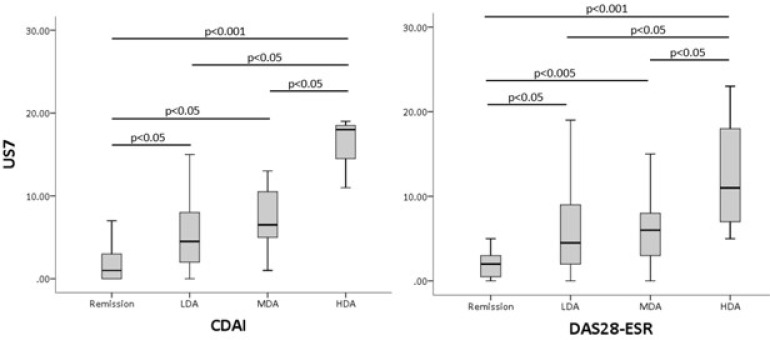
Associations between US7 score and disease activity in patients with rheumatoid arthritis according to A) CDAI B) DAS28-ESR.

The US7 scores were significantly different between group 1 and group 2 (p=0.021), but erosion scores were similar. The US7 scores in patients with HDA group were significantly higher than those in patients with remission, LDA, and MDA (p < 0.001, p < 0.05, p < 0.05 respectively). However, there were no significant differences in the US7 scores observed between patients with MDA and LDA groups (**Figure 2**). In addition, serum calprotectin levels were higher in those who had residual ultrasound disease activity than in those who fulfilled ultrasound remission criteria (102.3 ± 44.3 ng/ml vs 58.1 ± 38.2 ng/ ml, p=0.002).

When the patients with normal CRP levels were assessed (n = 34), serum calprotectin levels were found significantly higher in those with PD synovitis than those without PD synovitis (96.3 ± 45.7 vs 59.2 ± 44.7, respectively, p=0.024). When patients with normal ESR levels were assessed (n=31), considering calprotectin levels, there were found no statistical significance in patients with PD synovitis than those without PD synovitis (93.3 ± 50.3 vs 62.2 ± 47.3, respectively, p=0.086).

### Correlations between serum calprotectin levels and other disease activity parameters

The US7 score was positively correlated with calprotectin in patients with RA (r = 0.444, p < 0.001). Positive, higher, and statistically significant correlations were found between serum calprotectin levels and GS synovitis or GS tenosynovitis (r=0.441 and 0.360 respectively, p<0.001 for both assessments) (**Table 2**). In the cross-sectional analyses, serum calprotectin levels had a moderate to strong correlation with ESR, CRP, DAS28-ESR, DAS28-CRP, CDAI, SDAI, and HAQ (**Table 2**).

**Table 2.** Spearman’s rank correlation coefficients between calprotectin, US scores, and other variables.

	ESR	CRP	DAS28-ESR	DAS28-CRP	CDAI	SDAI	HAQ	Syn	Ten	PD syn	US7
Calprotectin	0.361^***^	0.306^**^	0.488^***^	0.495^***^	0.482^***^	0.429^***^	0.327^**^	0.441^***^	0.360^***^	0.359^***^	0.444^***^
ESR		0.494^***^	0.816^***^	0.561^***^	0.467^***^	0.554^***^	0.295^**^	0.271^*^	0.176	0.250^*^	0.247^*^
CRP			0.512^***^	0.780^***^	0.408^***^	0.923^***^	0.219	0.322^**^	0.234^*^	0.234^*^	0.309^**^
DAS28-ESR				0.828^***^	0.846^***^	0.695^***^	0.445^***^	0.431^***^	0.394^***^	0.400^***^	0.441^***^
DAS28-CRP					0.856^***^	0.924^***^	0.423^***^	0.484^***^	0.442^***^	0.408^***^	0.501^***^
CDAI						0.670^***^	0.507^***^	0.462^***^	0.500^***^	0.425^***^	0.509^***^
SDAI							0.342^**^	0.416^***^	0.343^**^	0.334^**^	0.418^***^
HAQ								0.351^***^	0.415^***^	0.257^*^	0.403^***^
Syn									0.671^***^	0.889^***^	0.964^***^
Ten										0.639^***^	0.832^***^
PD syn											0.870^***^

CDAI: clinical disease activity index; CRP: C-reactive protein; DAS28: disease activity score in 28 joints; ESR: erythrocyte sedimentation rate; HAQ: health assessment questionnaire; PD: power doppler; SDAI: simplified disease activity index; syn: ultrasound synovitis score; ten: ultrasound tenosynovitis score; US7: the 7-joint ultrasound score

*** *p* < 0.001,

** *p* < 0.01,

* *p* < 0.05

### The ability of calprotectin in predicting PD synovitis in patients with RA

**Figure 3.**
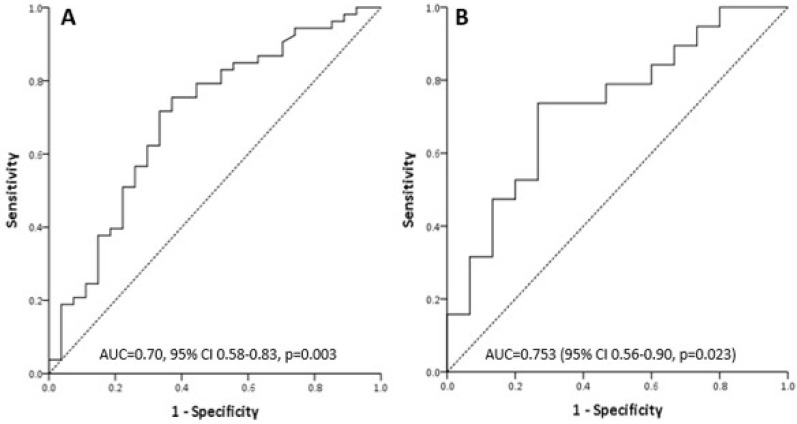
Receiver operating characteristics (ROC) curves for serum calprotectin level predicting disease activity when setting the Power doppler synovitis as the reference standard in rheumatoid arthritis according to A) assessment of whole RA group B) assessment of patients with normal CRP levels.

Optimal calprotectin cut-off levels for PD synovitis according to the assessment of the whole RA group are shown in **Figure 3A**. A cut-off point of 115.9 ng/ml had 50.9 % sensitivity, 77.8 % specificity, and area under curve (AUC) was 0.70 (95% CI 0.58–0.83, p=0.003). When we performed the same analyses in patients with normal CRP levels, the same calprotectin cut-off point of 115.9 ng/ml had 47.4% sensitivity, 86.7% specificity, and AUC was 0.75 (95% CI 0.56–0.90, p=0.023) (**Figure 3B**).

### Predictive value of calprotectin and CRP for PD synovitis in logistic regression analysis

Two separate models of regression analysis, which used the PD US synovitis as a dependent variable, were calculated with calprotectin and CRP. In each model, calprotectin and CRP were used one by one to avert probable collinearity (**Table 3**). Calprotectin, but not CRP, had a significant contribution to clarifying PD synovitis as a dependent variable in the logistic regression models (p = 0.024 in model 1 for calprotectin and p = 0.630 in model 2 for CRP). In the regression analysis, predicting the value of the calprotectin for PD synovitis was not affected by adding other parameters such as sex, age, duration of disease, and ACPA positivity, but the predictive value of CRP was not significant in multivariate analysis.

**Table 3.** Separate logistic regression analysis models for calprotectin and CRP to predict PD synovitis.

***Model 1***	**OR**	**95% CI**	**p**

Age	1.007	0.955 – 1.062	0.794
Gender	1.614	0.389 – 6.692	0.510
Disease duration	1.016	0.953 – 1.084	0.619
SJC	2.020	0.932 – 4.379	0.075
ACPA positivity	1.530	0.499 – 4.697	0.457
Calprotectin	**1.014**	**1.002 – 1.027**	**0.024**

***Model 2***	**OR**	**95% CI**	**p**

Age	1.006	0.955 – 1.060	0.818
Gender	1.327	0.345 – 5.101	0.681
Disease duration	1.018	0.958 – 1.083	0.562
SJC	2.347	1.033 – 5.332	0.042
ACPA positivity	1.119	0.385 – 3.251	0.837
CRP	1.009	0.974 – 1.045	0.630

## DISCUSSION

Rheumatoid arthritis is a widespread chronic inflammatory disease. Widely accepted clinical parameters developed by international groups have been used to assess disease activity in RA. C-Reactive protein and ESR are considered the best available laboratory markers of systemic inflammation but are not always associated with disease activity and progression. Uncomplicated methods are required to evaluate both RA activity and treatment effectiveness.

This study was conducted to determine the relation of serum calprotectin levels with clinical, laboratory, and ultrasonographic disease activity parameters in patients with RA. We studied 80 patients with RA and compared them with 30 healthy subjects, and calprotectin was significantly elevated in the RA group, particularly in patients who had moderate to severe disease activity. In addition, the US7 scores were significantly higher in patients with severe RA. These results indicate the importance of serum calprotectin levels and ultrasonographic assessment for the estimation of disease activity in RA patients. The results of the correlation analysis showed that there were statistically significant associations between calprotectin, and US7 and PD synovitis.

These findings were compatible with other studies that reported the correlation between MSUS findings and calprotectin.^17,18,26,27^ Our results were comparable with a recent study by Hurnakova et al., which evaluated 160 patients with RA.^17^ They suggested that calprotectin had an additional role in evaluating inflammatory activity in RA patients, and it was a better prognosticator of the RA activity, which was assessed with PDUS synovitis, than routinely used acute phase reactants. Similar to our results, their regression analyses showed that calprotectin had a better association with PDUS synovitis score than CRP. Inciarte-Mundo et al. evaluated the relationship between serum calprotectin levels and PDUS synovitis in RA and psoriatic arthritis (PsA) patients who were under tumour necrosis factor-alpha inhibitor treatment.^26^ They showed a more significant correlation between serum calprotectin and doppler and synovial hypertrophy scores than CRP and ESR. The present study also showed that calprotectin had a better correlation with US assessment parameters than with commonly used acute phase reactants. Nordal et al. also reported that serum calprotectin levels were significantly correlated with clinical, laboratory, and ultrasonographic activity parameters.^18^ According to their results, calprotectin was a potential indicator of inflammation in RA patients, and correlated better than the conventional inflammatory parameters. Hammer and colleagues have assessed the associations between serum calprotectin level and US evaluation during adalimumab treatment.^27^ Although they had a small number of patients, they found that calprotectin was associated with US assessment and sensitive to improvement in disease activity during adalimumab treatment in the 12-month follow-up. Jonsson et al. found a significant correlation, not only between calprotectin and US defined inflammation before and during DMARD treatment, but also in radiographic progression in patients with early RA.^19^

In some studies, it was shown that commonly used acute-phase reactants like CRP and ESR may not reflect the real disease activity, and they may remain stable in spite of the ongoing inflammation; furthermore, nearly 50% of patients with active RA have a normal CRP level.^28,29^ On the other hand, calprotectin is different from other acute phase reactants, because it is expressed in synovial tissue macrophages and activated synovial cells, and high levels of calprotectin were found in the synovial fluid of patients with RA,^5,10^ and precisely represents the total level of activated macrophages and reflect ongoing local inflammation. Therefore, calprotectin could be an auspicious indicator for inflammatory activity and has a probable superiority to CRP in RA.^30^ There is an increasing data supporting the importance of musculoskeletal US and its clinical usage in the evaluation of patients with RA, especially PD signal may predict future inflammatory activity in patients who are in remission or who have LDA,^31^ and Scire et al. showed the relationship between PD positive synovial hypertrophy and increased relapse risk.^32^ For these reasons, we assessed the predictive value of calprotectin among active RA patients, who had PD synovitis in the setting of normal CRP levels, and found that the increased calprotectin levels may show a higher disease activity status, which was evaluated by PD synovitis, in patients with RA with normal CRP levels. Our observation was compatible with the results from previous studies, in which RA patients with normal CRP levels were recruited.^20–22^ The most relevant finding of our study was that we propose an optimal cut-off level of 115.9 ng/ml, which can help for the differentiation of ultrasonographically active RA patients, not only from healthy controls, but also from RA patients with normal CRP levels. Our findings may help clinicians, who do not always have the opportunity and/or time to use the ultrasound in their clinical practice and have patients with suspicious inflammation despite normal acute phase reactants. However, our findings should be confirmed in further studies with more patients.

Our study had some limitations. Firstly, it had a cross-sectional nature, for this reason, the activity of the disease and radiographic progression could not be determined in long-term follow-up. Secondly, the study included patients who had mostly long-standing disease. Thirdly, the usage of concomitant DMARDs, steroids, and NSAIDs were not standardised due to the observational nature of the study. Fourthly, although the specificity of the cut-off level that we assessed was good, its sensitivity was relatively lower. Finally, the ultrasonographic evaluation was performed by only one sonographer, and thus inter-observer reliability could not be assessed, but the sonographer was unaware of the patients’ clinical situations, and this could be considered the strength of our study.

In conclusion, this study confirmed that serum calprotectin levels have a strong correlation with the US7 score and can efficaciously represent disease activity in patients with moderate and severe RA, particularly in patients who present with certain clinical signs but not accompanied by increased CRP.
